# NLRP3 Is Involved in Neutrophil Mobilization in Experimental Periodontitis

**DOI:** 10.3389/fimmu.2022.839929

**Published:** 2022-02-23

**Authors:** Banndith Cheat, Coralie Torrens, Asmaa Foda, Brigitte Baroukh, Jeremy Sadoine, Lotfi Slimani, Véronique Witko-Sarsat, Olivier Huck, Marjolaine Gosset, Jérôme Bouchet

**Affiliations:** ^1^ Université de Paris, Laboratory of Orofacial Pathologies, Imaging and Biotherapies URP2496, Montrouge, France; ^2^ Laboratoire d’Excellence INFLAMEX, Paris, France; ^3^ Université de Paris, Plateforme Imageries du Vivant, Faculté de Chirurgie Dentaire, Montrouge, France; ^4^ Université de Paris, INSERM U1016, CNRS UMR 8104, Institut Cochin, Paris, France; ^5^ Université de Strasbourg, Faculté de Chirurgie Dentaire, Periodontology, Strasbourg, France; ^6^ INSERM, UMR 1260, Regenerative Nanomedicine, Fédération de Médecine Translationnelle de Strasbourg (FMTS), Strasbourg, France; ^7^ Service de Médecine Bucco-Dentaire, AP-HP, Hôpital Charles Foix, Ivry-sur-Seine, France

**Keywords:** periodontitis, NLRP3 inflammasome, inflammation, neutrophils, *Porphyromonas gingivalis (P. gingivalis)*

## Abstract

The NLRP3 inflammasome is overexpressed in gingiva of periodontitis patients but its role remains unclear. In our study, we use a periodontitis mouse model of ligature, impregnated or not with *Porphyromonas gingivalis*, in WT or NLRP3 KO mice. After 28 days of induction, ligature alone provoked exacerbated periodontal destruction in KO mice, compared to WT mice, with an increase in activated osteoclasts. No difference was observed at 14 days, suggesting that NLRP3 is involved in regulatory pathways that limit periodontitis. In contrast, in the presence of *P. gingivalis*, this protective effect of NLRP3 was not observed. Overexpression of NLRP3 in connective tissue of WT mice increased the local production of mature IL−1β, together with a dramatic mobilization of neutrophils, bipartitely distributed between the site of periodontitis induction and the alveolar bone crest. *P. gingivalis* enhanced the targeting of NLRP3-positive neutrophils to the alveolar bone crest, suggesting a role for this subpopulation in bone loss. Conversely, in NLRP3 KO mice, mature IL-1β expression was lower and almost no neutrophils were mobilized. Our study sheds new light on the role of NLRP3 in periodontitis by highlighting the ambiguous role of neutrophils, and *P. gingivalis* which affects NLRP3 functions.

## Introduction

The activation of nucleotide‐binding leucine‐rich repeat protein 3 (NLRP3) inflammasome in inflammation-induced alveolar bone loss has emerged as an important mechanism in the pathogenesis of periodontitis (1, 2). This multiprotein signaling platform assembles after the activation of membrane or cytosolic receptors by pathogens or endogenic cell stress (3, 4). The priming of NLRP3 involves a diversity of receptors, including the Toll-like receptors 4 (TLR-4) which is the main surface receptor for bacterial LPS (5). Under activation, NLRP3 recruits an adaptor protein ASC which in turn binds to pro-caspase 1, allowing its cleavage in mature caspase-1 and its activation (3, 4). This active tripartite complex ensures the maturation of pro-inflammatory cytokines from the IL-1 family (i.e. IL-1β, IL-18) and the activation of gasdermin D, responsible for cell death by pyroptosis (4). Still, NLRP3 inflammasome role remains ambiguous as it could be either detrimental in some inflammatory diseases (6), while it can play a protective role in others, *via* the regulation of microbiota homeostasis (7). NLRP3 overexpression in gingival tissues of patients with periodontitis correlates with a high level of expression of IL-1β and IL-18 (1, 8, 9). Moreover, the presence of some inflammasome-related proteins in the saliva (NLRP3, ASC, and IL-1β) have recently been proposed as suitable biomarkers of the disease and may indicate cell death by pyroptosis (10).

Among the most common oral diseases, the very high global prevalence of periodontitis makes it a major public health problem, especially since periodontitis is associated with an increased risk of certain systemic diseases such as rheumatoid arthritis or atherosclerosis (11, 12). Periodontitis is a chronic inflammatory disease linked to a dysbiosis of the oral microbiome and characterized by the inflammation of the gum and irreversible destruction of the periodontal attachment (*i.e.* cementum, periodontal ligament and alveolar bone). The inflammation-induced alveolar bone loss associated with periodontitis is driven by the establishment of a highly pathogenic biofilm composed of commensal and pathogenic bacteria. The presence of a large amount of *Porphyromonas gingivalis (P. gingivalis)* is associated with the depth of the periodontal pocket and is predictive of further periodontal destruction in periodontitis (13). *P. gingivalis* is now considered as a keystone pathogen implicated in oral dysbiosis, responsible for the disruption of host tissue homeostasis leading to periodontal breakdown (14, 15). The destruction of alveolar bone in periodontitis involves an imbalance between osteoclasts and osteoblasts (16). Indeed, inflammation stimulates an increase in osteoclastogenesis, as well as a decoupling between bone resorption and reformation in which Receptor Activator of Nuclear Factor Kappa-β Ligand (RANKL) expression in tissue plays a major role (17).

The aim of the current study is to better understand NLRP3 implication during the onset of periodontitis using the ligature method with or without *P. gingivalis* in NLRP3 knock out (KO) mice (18–20). Our results show that NLRP3 KO mice present an increased alveolar bone resorption and soft tissue destruction, as compared to WT mice in the absence of *P. gingivalis*, suggesting a protective role for NLRP3. Notably, the role of NLRP3 is modulated in the presence of *P. gingivalis*. The increased periodontal expression of IL-1β in WT mice was accompanied by a sharp increase of polymorphonuclear neutrophils (PMN) recruitment to the connective tissue. These neutrophils formed a barrier near the sulcus and were also targeted along the alveolar bone crest, where they expressed increasing amount of NLRP3, depending on the presence or not of *P. gingivalis*. The present study will help to establish a link between neutrophils, inflammasome, IL-1β and the activation of osteoclasts driving bone resorption.

## Materials and Methods

### Mice Breeding

NLRP3 knock-out (KO) mice were generated on C57BL/6 genetic background by Martinon et al. (2006) and were obtained from Dr. JL Connat (INSERM 866, Université Bourgogne Franche‐Comté, Dijon, France). The transgene was maintained on a heterozygous background to prevent genetic drift by backcrossing them with the appropriate inbred parental strain (C57BL/6) every three generations. Throughout the experiments, NLRP3 KO and wild-type mice (WT) were maintained as separate lines (max. 6/cage) on a 12-h light/dark cycle and fed ad libitum. The behavior and reproduction of NLRP3 KO mice were undistinguishable from those of WT mice. To avoid any potential effects of estrogen, a total of 36 C57BL/6 male adult mice (8 to 12 weeks old) were used for the experiments.

### Bacteria Culture

ATCC 33277 *P. gingivalis* strain was obtained from Pr. M Bonnaure-Mallet (U1241, Université Rennes 1 ‐Bretagne, France) (21). ATCC 33277 *P. gingivalis* strain was sub-cultured on Schaedler agar plates with 5% of fresh sheep blood and vitamin K1 (BD Bioscience, Grenoble, France) for 4 to 5 days and then the black colonies were transferred aseptically into 10 mL of growth medium consisting of a suitable bovine brain heart infusion (3.7% BHI broth) and supplemented with 0.25% yeast extract, 0.1% hemin and 0.1% menadione. Bacteria were grown in anaerobic conditions at 37°C for 3 days using GasPak™ EZ anaerobic pouch systems (BD Bioscience).

### Periodontitis Induction

Experimental periodontitis was induced in 4 groups (6-10 mice per group) as already described (20): 2 groups of NLRP3 KO (+/- *P. gingivalis*) and 2 groups of WT (+/- *P. gingivalis*). Briefly, all mice were intraperitoneally anesthetized with a mixture of ketamine (80 mg/kg) and xylazine (10 mg/kg). Sterilized black braided 6.0 silk threads (Ethicon, Somerville, NJ, USA) soaked in the sterile culture medium, or incubated for 24 hours in culture medium containing *P. gingivalis*, was placed into the palatal sulcus of the first upper right molar. The left molar was left intact and used as control.

To facilitate the first ligature placement, a slight incision was made at the junction between the gum and the tooth. The ligature was then blocked with a drop of glass ionomer cement (Fuji 1, GC, Japan). The ligature was inspected and replaced without incision twice a week. In order to evaluate the influence of ligature placement on the progression of periodontal tissue destruction, ligature was maintained for 28 days. A swelling of the palatine mucosa was observed after the first week of induction, rendering the ligature easier to insert. Infected and non-infected mice were kept in separate cages to avoid cross infection. Notably, in most mice, the ligature was lost from the sulcus 72h after its placement.

### Microcomputed Tomography Acquisition and Analysis

Micro-CT analysis of living mice was performed before the induction of periodontitis (D0), at 2 weeks (D14), and at 4 weeks (D28), using a Quantum FX Caliper micro‐CT scanner (Life Sciences, Perkin Elmer, Villebon S/Yvette, France). Mice were anesthetized with isoflurane (induction at 3‒4% under an airflow of 0.8 L/min and 1.5%‒2% under 0.4 L/min); constant delivery of isoflurane was achieved *via* a nose cone connected to the scan platform. Acquisition parameters were a 10 Å~ 10mm field of view with a 20‐μm isotropic voxel size. X-ray source was fixed at 90 kV, 160 μA. Samples were submitted to a 360° rotation, and 3 min exposure time (22). Following the scan, tridimensional images were reconstructed, and the resulting images were re-oriented in a standardized manner by OsiriX software (Pixmeo, version 5.8.5; Bernex-Switzerland).

From reconstructed 3D images, we traced an axial section along the palatal vault. 3 perpendicular sections were chosen for the analysis. The first crossed the two centers of the palatal root of the first molars (right and left), and was framed by the other two sections with an increment of 200 µm mesial and distal, and a 2D image representing a frontal cut was extracted from each.

Alveolar bone resorption was analyzed on these 2D images, using Fiji software (23), after application of automatic moment-preserving thresholding (24), by measuring the distance between 2 anatomical landmarks: the alveolar bone crest (ABC) and the cement-enamel junction (CEJ), on a coronal section of the maxillary first molars. The measurements were performed using Fiji software (Wayne Rasband, version java 1.80-171; National Institute of Health, USA) at baseline (D0), at D14 and at D28. An increase of ABC-CEJ distance at D14 or D28 as compared to D0 is interpreted as indicative of alveolar bone resorption.

### Tissue Preparation

At days 28, all mice were euthanized under profound anesthesia (ketamine (80 mg/kg) and xylazine (10 mg/kg)). Blood was drawn out and intracardiac lavage (20 mL of PBS pH 7,4) was performed following by intracardiac perfusion with a fixative solution (10 mL) containing 4% paraformaldehyde in PBS (Electron Microscopy Sciences, USA). Maxillas were then dissected and post-fixed by immersion in the same fixative solution overnight at 4°C. After rinsing in PBS for 24 hours, the maxillas were processed by decalcification at room temperature for 4 weeks in 4.13% EDTA, pH 7.2, changed twice a week, with constant stirring. After extensive washing in PBS, the samples were dehydrated in increasing concentrations of ethanol (70%, 95%, 100%) and toluene (100%) and finally embedded in paraffin (Paraplast plus; Sigma–Aldrich, Merck, Darmstadt, Germany). Serial 7-µm-frontal sections of the maxilla were cut with a microtome and laid on glass slices for subsequent histological and immunological staining.

### Periodontal Tissue Histomorphometric Analysis

Paraffine-embedded maxilla sections were deparaffinized with toluene at room temperature during 30 min, rehydrated through ethanol 100%, 95% and deionized water during 30 min and stained with Masson’s trichrome. Slides were stained in hematein-aluminum solution (100 mM aluminum potassium, 3 mM hematein, 1% acetic acid) for 5 min, followed by rinsing in warm running tap water for 5 min and washed by distilled water. Next, they were stained in a ponceau fuchsin solution (0.03% acid fuchsin, 0.01% ponceau, 2% acetic acid) for 2 min and quickly rinsed in a 2% acetic acid solution for 2 min. The differentiation was carried out in orange G phosphomolybdic acid solution (2% orange G, 1.5% phosphomolybdic acid) for 5 min followed by 5 min rinse in a 2% acetic acid solution until the collagen loses its red color. Then, the stained sections were transferred into light green solution (0.1% fast green, 2% acetic acid) for 5min and rinsed in a 2% acetic acid solution for 5 min and washed in distilled water for 5 min. All colorants were filtered before use. The stained sections were dehydrated in 95% ethanol for 3 min, 100% ethanol for 3 min and cleared in toluene for 3 min. After dehydration, slides were mounted with distrene-plasticizer-xylene (DPX) resin (PanReacApplichem, Germany). Histological microscopy was performed using a Leitz DMRB microscope (Leica Microsystems) set for transmitted light illumination and equipped with a Sony DXC‐950 CCD camera. For histomorphometric evaluation, various *in situ* analyses were performed using the imaging software Fiji (23). The periodontal attachment level (AL) was measured as the distance in μm from the cement-enamel junction (CEJ) to the bottom of periodontal pocket defined as the first functional collagen fibers of the periodontal attachment. The pocket depth was measured between the top of gingival margin and the bottom of the periodontal pocket. The thickness of connective tissue was measured between the alveolar bone crest and the down growth of epithelium margin, the digitation closest to the tooth was chosen for measurement. The blood vessels were counted in a ROI of 0.23 mm^2^ corresponding to a reproducible rectangle drawn between tooth, alveolar bone crest and the midline of the palate, on computerized images obtained at x200 magnification.

### Antibodies

The list of antibodies used for immunolabelling as well as their conditions of use are listed in **Table 1**.

**Table 1:** List of primary and secondary antibodies used.

Antibody	Company	Ref N°	Specie/Clone	IF: concentration or dilution	IHC: concentration or dilution
***Primary antibodies***					
Pro-IL-1β	Abcam	ab9722	rabbit pAb	2 µg/mL	–
Mature IL-1β	Abcam	ab205924	rabbit pAb	–	1:50
TNF-α	Abcam	ab1793	mouse mAb (clone 52B83)	–	5μg/ml
RANKL	Abcam	ab216484	rabbit pAb	1:200	–
CtsK	Abcam	ab19027	rabbit pAb	–	1:400
CD45	SantaCruz	sc-53665	rat mAb (clone 30-F11)	4 µg/ml	–
Neutrophil marker	SantaCruz	sc-59338	rat mAb (clone NIMP-R14)	2 µg/ml	–
NLRP3	Adipogen	AG-20B-0014-C100	mouse mAb (clone Cryo-2)	5 µg/ml	–
***Secondary antibodies***					
Anti-rat (488)	Invitrogen	A11006	Goat pAb	1:300	–
Anti-rabbit (Texas Red)	Invitrogen	R6394	Goat pAb	1:300	–
Anti-mouse (Texas Red)	Invitrogen	R6393	Goat pAb	1:300	–
Anti-rabbit HRP	Dako	P0217	Pig pAb	–	1:200

For immunofluorescence and immunohistochemistry assays, primary antibodies were incubated over night at 4°C on samples, and secondary antibodies were incubated during 1h at room temperature.

### Immunohistochemistry and Immunofluorescence Quantifications

For immunohistochemistry staining of CtsK and TNF-α, sections were deparaffinized with toluene at room temperature during 30 min, rehydrated through ethanol 100%, 95% and deionized water during 30 min and antigen retrieval was performed with citrate buffer pH 6 at 95°C for 20 min. Slides were incubated with 3% H_2_O_2_ in deionized water for 30 min at 37°C in order to block the endogenous peroxidases. Sections were then washed in PBS and blocked in PBS with 5% bovine serum albumin (PBS–BSA) for 1 hour at room temperature to avoid non-specific binding. Samples were incubated after with primary antibody (CtsK, TNF-α, see **Table 1**) in PBS-BSA, overnight at 4°C in a moist chamber. Then the sections were rinsed and incubated in PBS-BSA with the corresponding secondary antibody diluted in blocking solution for 1 hour at room temperature (**Table 1**). For immunohistochemistry staining of mature IL-1β, the same antigen retrieval was performed. Sections were blocked in PBS with 5% normal goat serum, 1% BSA and 0.05% Tween-20, and incubated overnight at 4°C in a moist chamber with anti-IL-1β antibody diluted in the blocking solution (**Table 1**). Then, the same endogenous peroxidase blockade protocol was performed, followed by incubation with the corresponding secondary antibody diluted in blocking solution (**Table 1**). All slices were incubated with peroxidase-coupled avidin-biotin-complex (VECTASTAIN Elite ABC Kit, Vector Labs, USA), and staining was visualized using the 3,3ʹ‐diaminobenzidine tetrahydrochloride (DAB) chromogen. After dehydration, slides were mounted with DPX.

For immunofluorescence staining of pro-IL-1β, CD45, NLRP3, neutrophils and RANKL, the antigen retrieval was carried out as mentioned in the immunohistochemistry part. Sections were washed in PBS and saturated at room temperature for 1 hour with PBS-BSA 5%, 0.1% Triton-X-100, followed by an overnight incubation at 4°C with primary antibodies diluted in blocking solution (**Table 1**). Then the sections were rinsed and incubated in PBS-BSA with the corresponding fluorescent secondary antibody diluted in blocking solution for 1 hour at room temperature (**Table 1**). After washes in PBS-BSA, nuclei were stained with a solution of DAPI at 1 µg/mL (ThermoScientific, Germany). After rinsing in PBS and dehydration at room temperature for 5 min, coverslips were mounted on samples with 20 μL of Glycergel mounting medium (Dako, USA). In all immunostaining assays, negative controls were prepared by replacing the primary antibody with nonimmune serum or an irrelevant secondary antibody.

Acquisition of immunohistochemistry and immunofluorescence images was performed by microscopy as indicated in the histomorphometric analysis section. Quantitative analysis was performed on computerized images. For quantitative analysis, a ROI of 0.082 mm^2^ was selected in the connective tissue at the vicinity of the site of induction. Positive-stained cells were then counted manually in the ROI. We used Fiji software (23) to quantify the level of NLRP3 expression along the alveolar bone crest. A (130 µm length) x (10 µm thick) segmented line was manually drawn along the alveolar bone crest, and the mean intensity was measured by Fiji.

Co-staining analysis between NLRP3 and PMN (see **Table 1** for antibodies) was performed using Fiji software with the Colocalization Threshold plugin on a constant region cropped around the alveolar bone crest. Threshold was performed using the Costes method autothreshold determination (25). Mander’s and Pearson’s coefficients were calculated for the analysis.

Visualization of co-staining of neutrophils and NLRP3 was carried on a DMI6000 wide-field microscope (Leica) equipped with a Plan-Apochromat 63× objective. Images acquisition was done with Metamorph (Molecular Devices). Z-stack optical sections were acquired at 0.3-μm-depth increments. Deconvolution of complete image stacks was performed with Huygens Pro (version 14.10, Scientific Volume Imaging).

### Statistics

The statistical analysis was performed on Prism software (Graph Pad V.8). The differences were tested for significance, after normality verification with the Shapiro-Wilk test adapted to small samples. When normality was established for all groups, we used ordinary one-way ANOVA non-parametric test, followed by uncorrected Dunn’s test for the micro-CT analysis of bone resorption, and the uncorrected Fisher’s test for histomorphometric and immunostaining analyses. When normality was not established for one or more groups, Kruskall–Wallis test was applied, followed by group comparisons using Welch’s t‐test. The Spearman correlation coefficient (Spearman’s ρ, non-normal distribution) was used to determine the level of correlation between RANKL fluorescence intensity values and osteoclast numbers or ABC-CEJ distance. Unpaired Welch’s T test was used for the alveolar bone crest NLRP3 mean of fluorescence intensity analysis. P-values are represented as follows: ****P < 0.0001; ***P < 0.001; **P < 0.01; *P < 0.05; ^n.s.^P ≥ 0.05, non-significant.

### Study Approval

All experiments involving animals were performed in the animal facility of the Faculty of Dental Surgery (no. D 92‐049‐01) and received ethical approval from the French Ministry of Higher Education, Research, and Innovation (approval APAFIS no. 2020032719365322).

## Results

### NLRP3 KO Mice and *P. gingivalis*-Associated Experimental Periodontitis Display an Increased Level of *Alveolar* Bone Resorption

**Figure 1:**
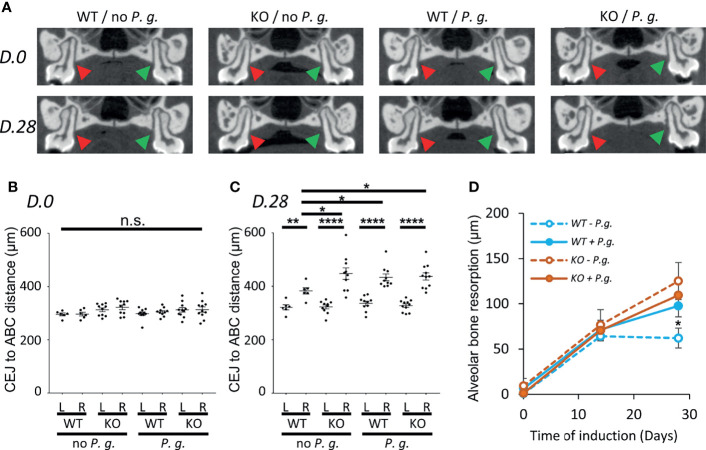
Alveolar bone resorption measurement on Micro-CT images. WT or NLRP3 KO mice were subjected to periodontitis induction with the insertion of a silk ligature with (*P. g.*) or without *P. gingivalis* (no P. g.) on the palatal side of the first right molar as described in the material and method section. Micro-CT analysis was performed before (D. 0), during (D. 14), and at the end of the experiment (D. 28). **(A)** The distance between Cement-Enamel Junction (CEJ) and Alveolar Bone Crest (ABC) was measured on 3 consecutive sections (100 µm) of the first palatine molars centered around the section shown in A, both on the left non-treated side (red arrowhead, L) and the right treated side (green arrowhead, R). **(B, C)** Mean of CEJ to ABC distances are shown, each dot represent the mean obtained from a single mouse. Measurement of D. 0 **(B)** and D. 28 **(C)** are shown, horizontal bars represent the mean +/- SEM. **(D)** Time course plots representing the mean +/- SEM of the difference between right (R) and left (L) side of the CEJ to ABC distance shown in **(B, C)**. Blue lines represent WT mice, orange lines represent NLRP3 KO mice, solid line are mice treated with *P. gingivalis*, dashed lines are mice treated without *P. gingivalis*. Uncorrected Dunn’s test. *P < 0.05, **P < 0.01, ****P < 0.0001, ^n.s.^P ≥ 0.05, non-significant.

Using high-resolution micro-CT, we performed a longitudinal follow-up measurement of the alveolar bone resorption (ABC-CEJ distance) in WT and NLRP3 KO mice in the absence or in the presence of *P. gingivalis*, before, during, and at the end of the experiment (D0, D14, D28) (**Figures 1A–D**). While no significant difference was observed initially among the 4 groups, and between the left (control) and right (ligature +/- *P. gingivalis.*) maxillary sides (**Figures 1A, B**), we could measure an increase of bone resorption starting at D14 on the right treated side but not at the level of the left molar, thus validating our experimental system. However, at this time point, no significant difference was observed between the 4 groups (**Figure 1D**). After 28 days of induction, no significant increase was detected in WT mice treated in the absence of bacteria (-3.3%, compared to D14), indicating that the periodontal destruction is stabilized as it has been already described (20). In contrast, in NLRP3 KO mice, the bone resorption continues to progress between day 14 to day 28 (+26.6%, compared to D14) suggesting that NLRP3 could play a role in the regulatory mechanisms that limit trauma-induced periodontal lesion.

With regards to infection, the insertion of *P. gingivalis* in the gingival crevice significantly increased bone resorption (+38.8% compared to D14) at Day 28 in WT mice as compared to uninfected mice (**Figures 1C, D**). On the opposite, we could not observe any cumulative effect of the absence of NLRP3 and the treatment with *P. gingivalis* in KO mice as alveolar bone resorption was similarly increased (+35.6%, compared to D14) (**Figures 1C, D**). From this data, we can conclude that the expression of NLRP3 in mice has a protective effect on induced periodontal bone loss only in the absence of infection.

### NLRP3 Shows a Protective Effect Against Connective Tissue Loss of Attachment, and Is Subverted by the Presence of *P. gingivalis*

**Figure 2:**
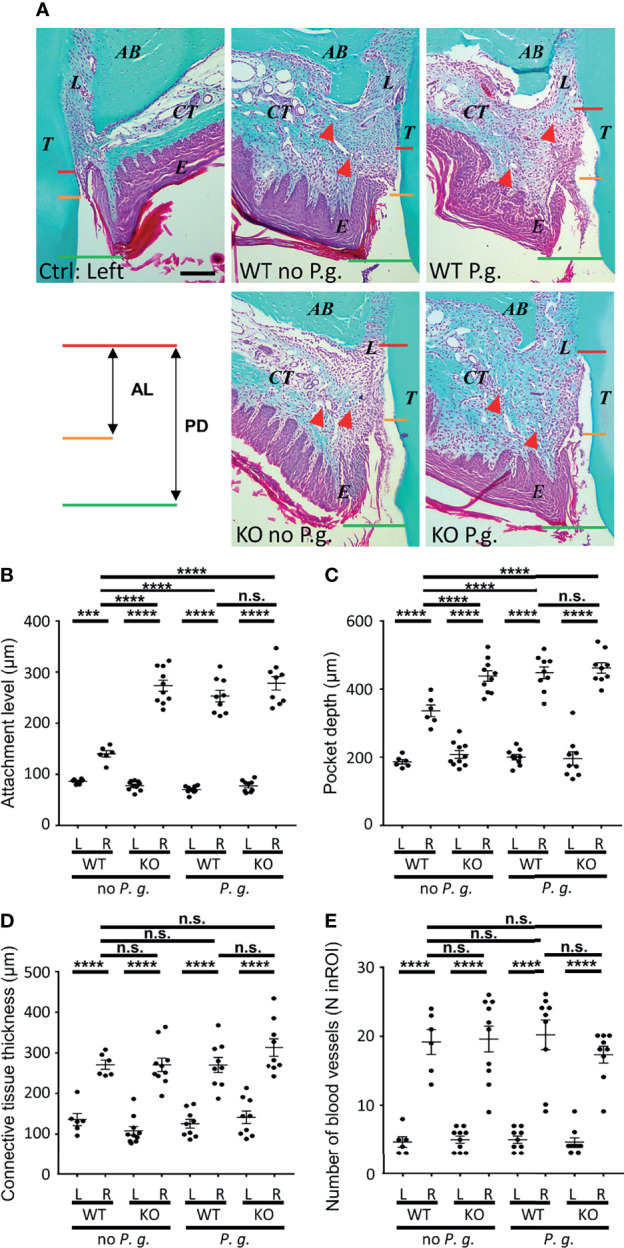
Histological characteristics of periodontal region after periodontitis induction. **(A)** Representative images of Masson Trichrome-stained sections of periodontitis side (right panels) and control side (individual left panel) at 10X objective lens magnification. (T, tooth, first palatine molar; L, periodontal ligament; AB, alveolar bone; CT, connective tissue; E, epithelium; the red triangular indicates the blood vessel). The measurements of pocket depth (PD, between red line and green line) and the attachment level (AL, between red line and yellow line) are shown. Scale bar = 200μm. **(B)** The attachment level (AL) is the distance measured from cemento-enamel junction (**A**, yellow lines) to the bottom of the periodontal pocket (**A**, red lines). **(C)** The pocket depth (PD) is the measured distance from the gingival margin (**A**, green lines) to the bottom of the sulcus (**A**, red lines). **(D)** The connective tissues thickness was measured from the alveolar bone crest to the base of epithelium. **(E)** The blood vessels were counted in a standardized region of interest (ROI) of the connective tissue (ROI = 0.23 mm^2^). **(B–E)** Each dot represents the measurement obtained from a single mouse. Horizontal bars represent the mean +/- SEM. Ordinary one-way ANOVA followed by uncorrected Fisher’s LSD, with a single pooled variance multiple comparison test. ***P < 0.001, ****P < 0.0001, ^n.s.^P ≥ 0.05, non-significant.

Histological analysis of mice periodontium revealed a deepening of the periodontal pocket (from the gingival margin to the bottom of the sulcus), together with an increase of the attachment loss (CEJ to sulcus bottom) after induction with or without *P. gingivalis* (**Figures 2A–C**). WT mice treated without *P. gingivalis* showed a slight increase of the periodontal pocket depth (**Figure 2B**), as well as a minor loss in the level of attachment as compared to the control side (**Figure 2C**). In comparison, KO mice treated without *P. gingivalis* showed a deeper periodontal pocket, accompanied by an increased loss of attachment level, supporting the protective role for NLRP3 expression in WT mice. WT mice treated with *P. gingivalis* also showed significantly increased pocket depth and attachment loss profiles, compared to non-*P. gingivalis*-treated WT mice. These features are similar to those of KO mice treated without *P. gingivalis*, and we observe no cumulative effect of *P. gingivalis* periodontitis-induction and the lack of NLRP3 expression in KO mice with *P. gingivalis* (**Figures 2B, C**).

### General Features of Inflammation Are Not Dependent on NLRP3 Expression or the Presence of *P. gingivalis* in the Periodontal Lesion

We first focused on analysis of connective tissue thickness and blood vessels (number, diameter) as markers of gingival inflammation using Masson’s Trichrome staining. Comparison of the connective tissue thickness between the different conditions (WT or NLRP3 KO mice, treated or not with *P. gingivalis*) revealed similar swelling on the treated right side, as compared to the left side (**Figure 2D**). Likewise, the number of blood vessels was largely increased in connective tissue of the right side, with no difference observed among the 4 groups (**Figure 2E**).

**Figure 3:**
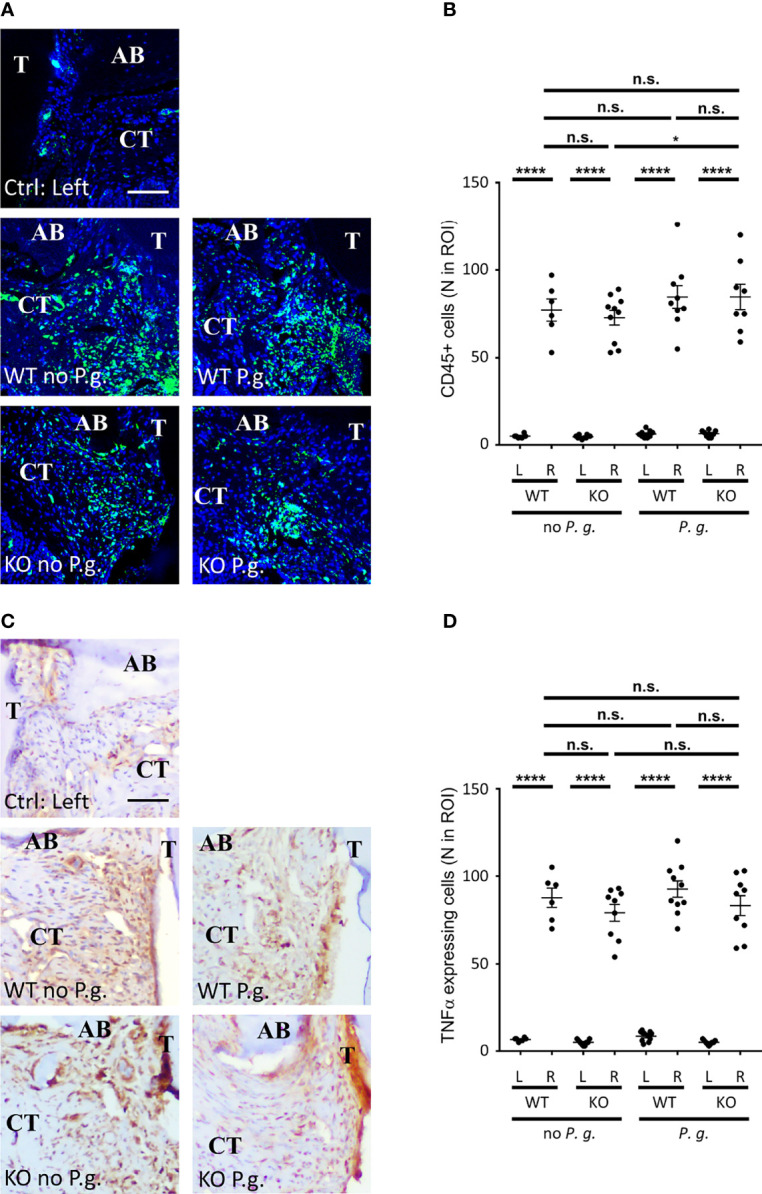
CD45+ cells and TNF-α expression in connective tissue. **(A)** CD45 expression was revealed by immunofluorescence. **(B)** The number of CD45^+^ cells in a standardized ROI (ROI = 0.082 mm^2^) was quantified. **(C)** TNF-α expression was revealed by immunohistochemistry. **(D)** The number of TNF-α-expressing cells (brown staining) in a standardized ROI (ROI = 0.082 mm^2^) was quantified. **(A, C)** The control without periodontitis induction (left side) is shown on the individual upper panel, the assays with ligature with or without *P. gingivalis* (right side) are shown on the 4 lower panels (CT = connective tissue, AB = alveolar bone), scale bars = 50 μm. **(B, D)** Each dot represents a single mouse. Horizontal bars represent the mean +/- SEM. Ordinary one-way ANOVA followed by uncorrected Fisher’s LSD with a single pooled variance multiple comparison test. *P < 0.05, ****P < 0.0001, ^n.s.^P ≥ 0.05, non-significant.

To get further insight into the characterization of inflammation, we quantified the concentration of CD45^+^ leucocytes as a marker of the inflammatory cells infiltrate in the connective tissue (Pan-CD45 staining, see **Table 1**). Ligatures +/- *P. gingivalis* triggered a similar recruitment of CD45^+^ cells among the 4 different groups in the vicinity of the treated sulcus (**Figures 3A, B**). Of note, a significant 16%-increase of CD45^+^ cells infiltration was measured between KO mice treated or not with *P. gingivalis*. The presence of the bacteria may be responsible for this light increase in KO mice. In parallel to the recruitment of leukocytes, the amount of the pro-inflammatory cytokine TNFα was similarly increased in the right-treated side of all groups (**Figures 3C, D**). Together with the histological analyses, our results indicate a strong induction of the local inflammation adjacent to the site of periodontitis in our mouse model, with a similar intensity among the 4 different groups.

### RANKL Expression and Osteoclast Activity Reflect the Level Alveolar Bone Resorption

**Figure 4:**
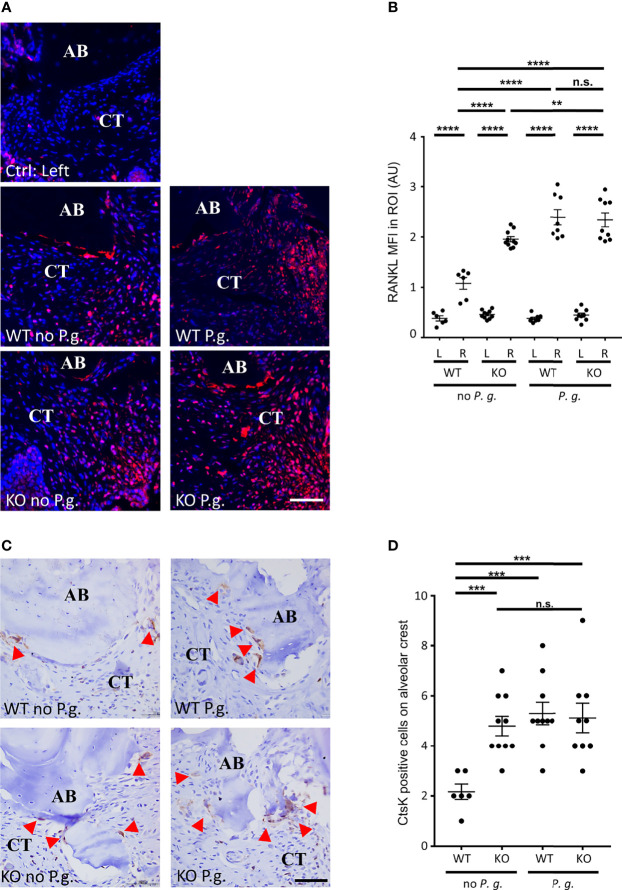
Active osteoclasts and RANKL expression. **(A)** The expression of RANKL was revealed by immunofluorescence. The control without periodontitis induction (left side) is shown on the individual upper panel, the assays with ligature with or without *P. gingivalis* (right side) are shown on the 4 other panels (CT = connective tissue, AB = alveolar bone). **(B)** RANKL mean of fluorescence intensity (MFI) was measured in a 0.082 mm^2^ area (ROI) surrounding the alveolar bone crest. **(C)** Active osteoclasts were revealed by immunohistochemistry targeting the active osteoclasts marker Cathepsin K (Ctsk). Red arrowheads show Ctsk^+^ osteoclasts. Only the right sides of treated mice are shown (CT = connective tissue, AB = alveolar bone). **(D)** The number of Cathepsin K-positive cells was counted in a 0.082 mm^2^ area (ROI) surrounding the alveolar bone crest. Scale bars = 50μm. **(B, D)** Each dot represents a single mouse. Horizontal bars represent the mean +/- SEM. Ordinary one-way ANOVA followed by uncorrected Fisher’s least significant difference (LSD) with a single pooled variance multiple comparison test. **P < 0.01, ***P < 0.001, ****P < 0.0001, ^n.s.^P ≥ 0.05, non-significant.

As RANKL plays a key role in the differentiation and activation of osteoclasts (26), we quantified the level of RANKL expression in connective tissue near the alveolar bone crest. As suspected, we measured an increase in the expression of RANKL in the connective tissue of treated right side in all groups, compared to the untreated left side (**Figures 4A, B**). Interestingly, in WT mice without *P. gingivalis*, the increase in RANKL expression was less than in the three other groups. Of note, we measured a small but significant difference between KO mice without *P. gingivalis* and *P. gingivalis*-treated mice.

To analyze involvement of osteoclasts in alveolar bone resorption, we used a Cathepsin K (CtsK) staining to quantify the number of active multinucleated osteoclasts along alveolar bone (**Figures 4C, D**). This cysteine protease is involved in osteoclastic bone resorption and is considered to be a specific marker for active osteoclasts (27). We found multinucleated CstK-positive cells on the alveolar bone surface on the right side of all treated mice, whereas we found virtually no osteoclasts on the bone surface on the untreated side (data not shown) (**Figure 4C**). Yet, the number of osteoclasts on the alveolar bone surface of WT mice treated with ligature alone (absence of *P. gingivalis*) was significantly less increased (50%) compared to the other three conditions (**Figure 4D**).

Notably, we found that the level of RANKL expression shows a relationship with the level of alveolar bone resorption (moderate correlation ρ = 0.485, P = 0.004) (**Figure 1**) and with the osteoclast activity (weak correlation ρ = 0.386, P = 0.027) (**Figures 4A, B**).

### The Presence of *P. gingivalis* Increases NLRP3 Expression Within Periodontal Tissues

**Figure 5:**
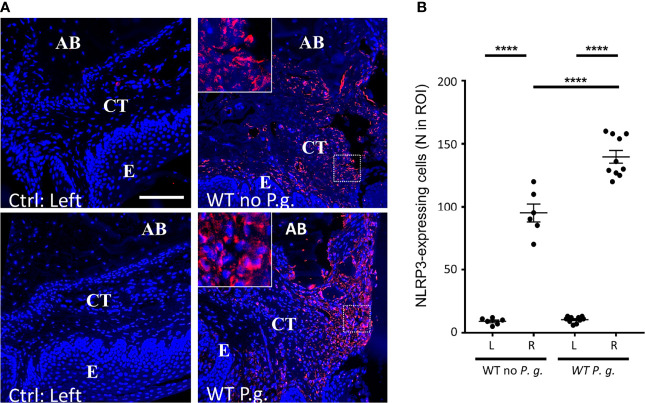
NLRP3 is overexpressed in periodontitis connective tissue of WT mice. **(A)** NLRP3 protein expression was revealed by immunofluorescence. The control without periodontitis induction (left side) are shown on the left panels, the assays with ligature with or without *P. gingivalis* (right side) are shown on the right panels. The square enlargement helps to visualize individual cells along alveolar bone. (CT = connective tissue, AB = alveolar bone, E = epithelium), scale bar = 100 μm. **(B)** The number of NLRP3-expressing cells in a standardized ROI (ROI = 0.082 mm^2^) was quantified. Each dot represents a single mouse. Horizontal bars represent the mean +/- SEM. Ordinary one-way ANOVA followed by uncorrected Fisher’s LSD with a single pooled variance multiple comparison test. ****P < 0.0001.

We quantified the number of cells expressing NLRP3 in connective tissue of WT animals. As expected, in NLRP3 KO mice, no expression of NLRP3 was detected (not shown), confirming the specificity of NLRP3 antibody, and as previously described (18). In WT mice, while the non-treated connective tissue virtually did not show any detectable NLRP3 expression, we could evidence a strong NLRP3 expression on the right side, treated with ligature alone or with *P. gingivalis* (**Figure 5A** + enlargement). This result is reminiscent with the upregulation of NLRP3 in periodontitis patient gingiva described elsewhere (1, 8, 10). Interestingly, the number of NLRP3 expressing cells was significantly increased when mice were treated with *P. gingivalis* (**Figure 5B**).

### NLRP3 Expression Increases IL−1β Expression

**Figure 6:**
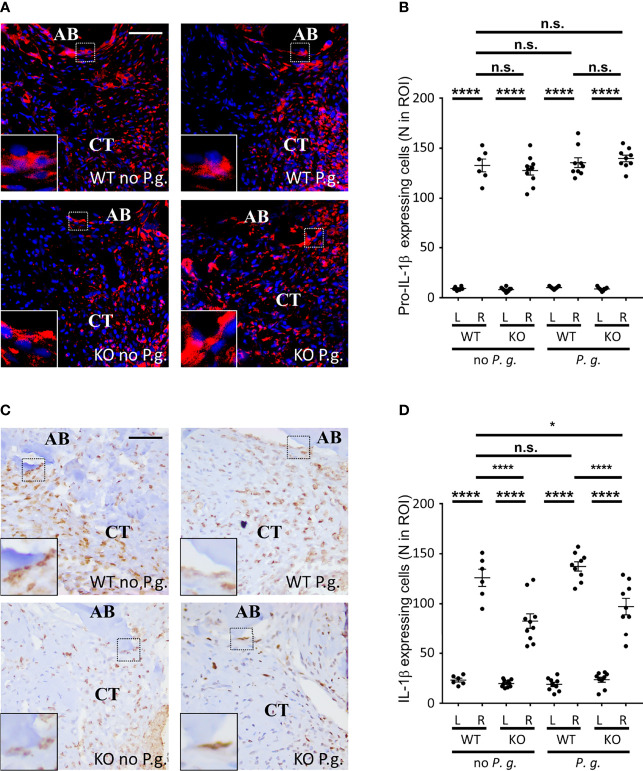
NLRP3 expression is necessary for an optimal IL-1β cleavage. **(A)** Pro-IL-1β protein expression was revealed by immunofluorescence using a specific antibody targeting the immature form. Enlargements show cells expressing pro-IL-1β along alveolar bone. **(B)** The number of pro-IL-1β-expressing cells was counted in a 0.082 mm^2^ area (ROI) surrounding the alveolar bone crest. **(C)** IL-1β protein expression was revealed by immunofluorescence using a specific antibody targeting the mature form. Enlargements show cells expressing IL-1β along alveolar bone. **(D)** The number of mature IL-1β-expressing cells was counted in a 0.082 mm^2^ area (ROI) surrounding the alveolar bone crest. **(B, D)** Each dot represents a single mouse. Horizontal bars represent the mean +/- SEM. Ordinary one-way ANOVA followed by uncorrected Fisher’s LSD with a single pooled variance multiple comparison test. *P < 0.05, ****P < 0.0001, ^n.s.^P ≥ 0.05, non-significant.

As NLRP3 inflammasome activation and assembly results in the maturation of pro-IL-1β into mature IL−1β (4), we measured the expression of mature IL−1β in the connective tissue of WT and KO mice. The expressions of both pro-IL-1β and its mature form were barely detectable on the left control side (**Figures 6B, D** and **Supplementary Figures S1A, B**). On the ligature-treated right side, the level of pro-IL-1β-expressing cells was strongly increased with no significant difference among the 4 groups, showing that the trauma induced by ligature is sufficient to trigger the expression of pro-IL-1β in cells (**Figures 6A, B**). As expected, we could detect a stronger expression of the mature form of IL-1β in WT mice than in NLRP3 KO mice, (**Figures 6C, D**). Importantly, for each NLRP3 KO mice we observed a decrease of the number of cells expressing mature IL−1β, compared to the number of cells expression pro-IL-1β, regardless of the treatment with or without *P. gingivalis*, indicating that the overpassing of NLRP3 by *P. gingivalis* infection is not a direct mechanism (**Supplementary Figure S1C**). Moreover, the ratio between cells expressing pro-IL−1β and mature IL−1β was close to 1 in tissues from WT mice expressing NLRP3, indicating a full activity of inflammasomes in WT mice, while this ratio decrease to ~0.65 in KO mice, therefore revealing the importance of NLRP3 inflammasome in the production of mature IL−1β in these mice (**Supplementary Figure S1D**).

### Neutrophils Differentially Infiltrate Periodontal Tissue in WT Versus NLRP3 KO Mice

**Figure 7:**
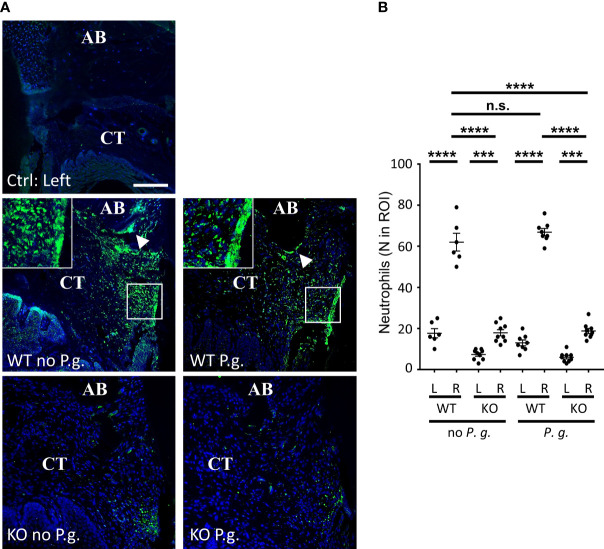
Neutrophils infiltrate connective tissue dependently of NLRP3 expression. **(A)** Neutrophils were detected by immunofluorescence using a specific antibody. The control without periodontitis induction (left side) is shown on the individual upper panel, the assays with ligature with or without *P. gingivalis* (right side) are shown on the 4 lower panels. Enlargements show the “Neutrophil wall”, close to the site of periodontitis induction. White arrowheads show neutrophils along alveolar bone (CT = connective tissue, AB = alveolar bone), scale bar = 100 μm. **(B)** The number of neutrophils was counted in a 0.082 mm^2^ area (ROI) surrounding the alveolar bone crest. Each dot represents a single mouse. Horizontal bars represent the mean +/- SEM. Ordinary one-way ANOVA followed by uncorrected Fisher’s LSD with a single pooled variance multiple comparison test. ***P < 0.001, ****P < 0.0001, ^n.s.^P ≥ 0.05, non-significant.

As Polymorphonuclear Neutrophils (PMN) are thought to be the most efficient phagocyte to destroy bacteria during periodontal infection (28), we examined whether their presence in the connective tissue neighboring the site of periodontitis induction will depend on NLRP3 or on the presence of exogenous *P. gingivalis* (**Figure 7**). Although the time point of 28 days that we used cannot be considered as an early phase of the trauma, we found a strong recruitment of PMN in the vicinity of the periodontal lesion in WT mice, in the absence of *P. gingivalis* by using a specific staining of PMN on periodontal histological section (**Figures 7A, B**). By contrast, periodontal injury of NLRP3 KO mice, in the absence of *P. gingivalis* did not induce any increase in PMN recruitment in the adjacent tissue, strongly suggesting a defect for of PMN targeting in NLRP3 KO mice (**Figures 7A, B**).

With regards to the infection with *P. gingivalis*, we could not detect any significant difference in neutrophil content within the periodontal tissues between WT mice treated or not with *P. gingivalis*, despite a significant effect on bone resorption (**Figure 1**).

Periodontal injury of NLRP3 KO mice, in the presence of *P. gingivalis* did not induce any increase of PMN content in the adjacent tissue, showing a defect of PMN content in KO mice similarly to what was observed in the absence of infection with *P. gingivalis* (**Figures 7A, B**).

A deeper analysis of PMNs in the connective tissue of WT mice revealed an inhomogeneous distribution of these cells within the tissue, independently of the presence of *P. gingivalis*. Indeed, while PMN recruitment was largely increased throughout the connective tissue surrounding the injured area, they were even more concentrated at the surface of alveolar bone crest (**Figure 7A**, arrowheads) and along the gingival crevice (**Figure 7A**, enlargements), here forming a true “PMN wall” surrounding the lesion and reminiscent of previous observations (28). This bipartite distribution was not observable in KO mice, as the number of PMN was significantly lower than in WT mice, even if the majority of PMN observed in KO mice appeared to be located around the site of periodontitis induction, regardless of the presence or not of *P. gingivalis*.

### NLRP3 Is Expressed Differentially in Neutrophils, Depending on the Presence of *P. gingivalis* in Periodontal Tissues

**Figure 8:**
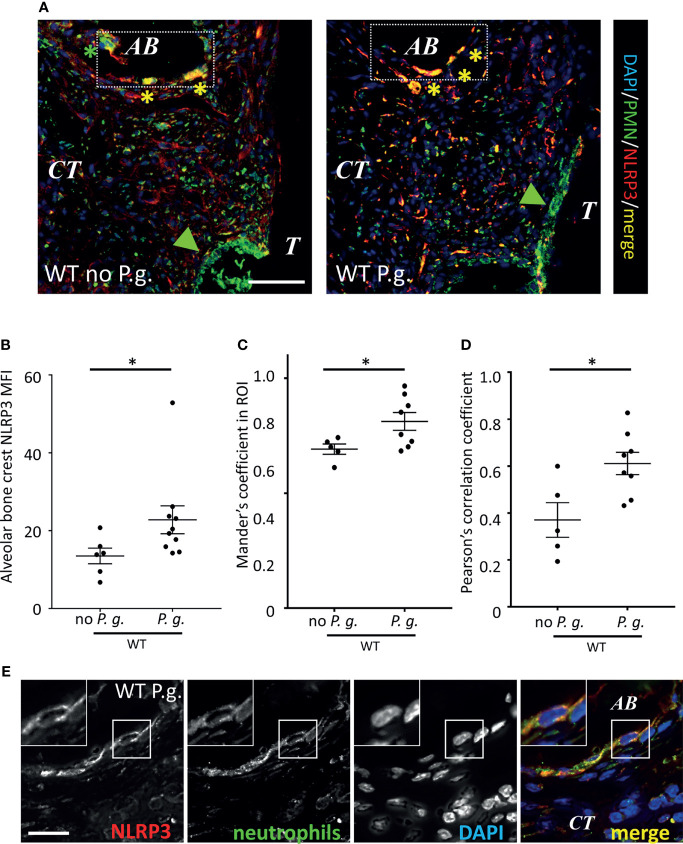
Neutrophils recruited around alveolar bone express NLRP3. **(A)** Neutrophils and NLRP3 were detected by immunofluorescence using specific antibodies. Bar = 100 μm. **(B)** NLRP3 mean of fluorescence intensity (MFI) was measured in a (130 µm length) x (10 µm thick) segmented line surrounding the alveolar bone crest. Green arrowheads show sulcular neutrophils, yellow asterisks show NLRP3-expressing alveolar bone neutrophils **(C, D)** Population analysis of the co-expression between NLRP3 and neutrophil marker. Mander’s **(C)** and Pearson’s **(D)** coefficients are shown. **(B–D)** Each dot represents a single mouse. Horizontal bars represent the mean +/- SEM. Unpaired Welch’s T test. *P < 0.05. **(E)** Enlargement shows a single neutrophil close to alveolar bone (AB) expressing NLRP3 in WT mice treated with *P. gingivalis*. Bar = 10 μm.

We measured a different expression of NLRP3 in PMN, depending on their localization in connective tissue. While NLRP3 was strongly expressed in PMN close to the alveolar bone (**Figure 8A**, rectangle), its expression was weaker in the periodontal pocket (**Figure 8A**, green arrowheads), suggesting different levels of PMN activation in the model, that could be either involved in protection, reparation or inflammation (**Figure 8A**). As NLRP3 appeared to be accumulated at the surface of alveolar bone (**Figure 5A**, **Figure 8A**, arrowheads), we measured the mean fluorescence intensity of NLRP3 around the alveolar bone crest. When mice were treated with *P. gingivalis*, the level of NLRP3 expression was 68% higher than the mice treated with ligature alone (**Figure 8B**), suggesting a specific role for NLRP3 in alveolar bone resorption induced by *P. gingivalis*. Moreover, we could measure an increase of NLRP3 expression in PMN against the alveolar bone crest from WT mice treated with *P. gingivalis*, as both Pearson’s and Mander’s coefficients between NLRP3 and PMN staining were increased in this condition (**Figures 8C, D**). It should be noted that the increased magnification of the neutrophils of the alveolar bone crest shows that the increase in the Pearson’s and Mander’s coefficients is not the result of a colocalization of the intracellular staining of PMN and NLRP3, but of a coexpression in cells (**Figure 8E**).

## Discussion

The present study describes a dual role for NLRP3 in bone resorption through the modulation of neutrophils recruitment into the periodontium. In the absence of *P. gingivalis*, NLRP3 is directly involved both in a significant limitation of osteoclast activation and in a strong neutrophil presence within the periodontal tissue to promote the limitation of bone resorption. In the presence of the bacteria, NLRP3 expression is again associated with a limitation of neutrophil presence within the periodontal tissue but this is not associated with the limitation of bone resorption, presumably because of a different “pro-inflammatory” phenotype of neutrophils.

Our results show an alveolar bone destruction induced by a permanent periodontal lesion in the gingival crevice of C57BL/6 mice, exacerbated by the presence of *P. gingivalis* and coinciding with the presence of active osteoclasts on alveolar bone. Comparable results were previously obtained by other groups, therefore validating our experimental system (20, 29, 30). In our hands, in WT mice without *P. gingivalis*, bone destruction reached a maximum after 14 days, suggesting the activation of a protective mechanism against uncontrolled damage, and allowing periodontal tissue to adapt to the lesion. Interestingly, Marchesan et al., showed a reduction of alveolar bone resorption after 9 days, in a mouse model of periodontitis, induced by a ligature coated with a variety of bacteria, but no *P. gingivalis*. *P. gingivalis* circumvents NLRP3 protection, because in its presence, bone resorption continued for up to 28 days.

In agreement, Lin et al. observed a mild resorption after 2 weeks of ligature with no effect of *P. gingivalis*, while alveolar bone loss was increased after ligature removal in the *P. gingivalis*-pretreated group only (31). In KO mice, we found that bone destruction was also increased between day 14 and day 28, again showing a loss of protection against uncontrolled inflammation in these mice and suggesting that this phenomenon is partially independent of the inflammatory process induced either by trauma only or by the presence of endogenous bacteria from the plaque in the area of periodontitis induction. This increase of periodontal tissue destruction in KO mice clearly shows a protective effect of NLRP3. Moreover, the lack of cumulative effect between *P. gingivalis*-induced bone resorption and the absence of NLRP3 protection in KO mice, suggests that the *P. gingivalis*-mediated alveolar bone destruction is dependent on NLRP3 expression, whereas an independent mechanism would have shown a cumulative effect in *P. gingivalis*-treated KO mice. *P. gingivalis* therefore reveals a pathologic activity of NLRP3 on alveolar bone resorption during periodontitis.

NLRP3 inflammasome contribution to alveolar bone loss in periodontitis has been studied in other experimental systems using various KO NLRP3 mice on C57Bl/6 genetic background. Subgingival injection of inactivated *Aggregatibacter actinomycetemcomitans*, did not show any significant role of NLRP3 or Caspase-1 in periodontal inflammation and bone resorption *in vivo* (32). In addition to the bacterial strain used, this model presents strong differences of experimental design with ours, which may explain these discrepancies. In a model of oral gavage, WT C57BL/6 mice were prone to alveolar bone loss after exposure to *P. gingivalis*, but no resorption was observed in NLRP3 KO mice (33). However, the genetic background of mice is crucial for periodontal destruction in the gavage model of periodontitis and C57BL/6 is not sensitive to this model. Indeed, de Molon et al. showed that oral gavage was not efficient in inducing periodontal inflammation and bone resorption in C57BL/6 compared to the ligature model (30, 33). Therefore, *A. actinomycetemcomitans.* injection and *P. gingivalis.* gavage models are not optimal models of periodontitis pathogeny in C57BL/6 mice, as they do not reproduce the formation of periodontal pockets nor the loss of attachment (32–34).

Globally, the general inflammation features we analyzed in mice were similar, regardless of NLRP3 expression or the presence or absence of *P. gingivalis*. A high level of inflammation is probably maintained in our experimental conditions, in which foreign material is left in the palatine gingival crevice during 28 days and may lead to the accumulation of bacteria in the injured area. Under these conditions, the presence of *P. gingivalis* appears to be secondary in the induction of periodontal inflammation. Yet, immunohistological analysis on WT mice revealed a local overexpression of NLRP3. The overexpression of NLRP3 in gingival tissue has been proposed as a negative factor in the evolution of periodontitis (32, 35, 36). Here we show that this increased expression is significantly exacerbated in the presence of *P. gingivalis*, showing that both ligature and *P. gingivalis* influence the regulation of inflammasome expression. The balance between bone resorption and repair in periodontitis could therefore result from fine-tuning of the NLRP3 inflammasome, the overactivation or absence of which could lead to pathologic bone loss.

Although pro-IL-1β was expressed independently of the expression of NLRP3, the number of cells positive for the mature IL-1β was significantly increased in WT mice, showing the functionality of NLRP3 inflammasome. Surprisingly, we could not detect any significant difference in expression of mature IL−1β; whether the WT mice were treated with *P. gingivalis* or not. It is likely that the level of pro-IL−1β reaches a maximum under our experimental conditions, and that the lower level of NLRP3 expression in WT mice without *P. gingivalis* is sufficient to ensure the optimal maturation of IL−1β. However, the remaining production of mature IL−1β in NLRP3 KO mice suggests that a fraction of the pool of IL−1β may be cleaved even in the absence of NLRP3, possibly by another inflammasome complex.

The infiltration of neutrophils in connective tissue and the formation of the characteristic neutrophil wall in the area of periodontal lesion was dependent on the expression of NLRP3. Likewise, the default of neutrophil recruitment to sites of inflammation has been observed in a mouse model of hepatic ischemia-reperfusion injury, where neutrophil targeting to the liver of NLRP3 KO mice was significantly reduced, as compared to WT mice (37). IL-1β is described to be an inducer of CXC-family chemoattractant cytokine expression in tissue (i.e. CINC-1/IL-8 and MIP-2/CXCL2), leading to the accumulation of neutrophils in the site of injury (38, 39).

Regarding the decrease of mature IL-1β production in NLRP3 KO mice, we propose that the recruitment of neutrophils may require a sufficient release of mature IL-1β in periodontal tissue to induce their chemoattraction. The recruitment of neutrophils to the site of periodontitis has long been described and their protective or destructive effect on periodontal tissue during periodontitis is still a matter of debate (40–42).

Although these cells are considered as the first line of defense during periodontal infection and inflammation, their concentration correlates with the severity of periodontitis (28, 43). Therefore, neutrophils are considered as the most prominent cells in periodontitis and they are found in a hyperactive state (44). By contrast, patients with defective neutrophils rare diseases display severe periodontitis that highlights the key role of neutrophils in periodontitis. Thus, patients with a reduction in their number (neutropenia) but also their function (e.g.: Papillon-Lefèvre syndrome) or their migration (e.g.: LAD-1 leukocyte adhesion deficiency syndrome) present a severe and early form of the disease (45, 46). We propose here NLRP3 as a regulator of neutrophil function, since its expression and activation regulate their accumulation in periodontitis resulting in a net beneficial effect on bone resorption suggesting a potential to modulate osteoclast activities.

Besides the neutrophil wall, we also observed in WT mice a concentration of neutrophils, close to the alveolar bone crest, some of them being directly in contact with the bone surface. These NLRP3-expressing neutrophils were at the front line of the bone remodeling site, close to the osteoclasts, where they may interact together. Close links have been established between neutrophil activation and physiological or pathological bone remodeling (45). For example, in chronic gout disease, activated neutrophils can provoke the retraction of osteoblasts and activate osteoclasts (47). Direct interactions between neutrophils and osteoclasts or osteoblasts are therefore conceivable. Chakravarti et al. described *in vitro* that an upregulation of RANKL expression at the surface of TLR-4-stimulated neutrophils of patients with rheumatoid arthritis lead to osteoclast activation and subsequent bone resorption (48). Similarly, in our experiments, stimulation of neutrophils by *P. gingivalis* in WT mice could increase the expression of NLRP3 and RANKL, activate osteoclasts and improve alveolar bone resorption. The differential expression of NLRP3 in neutrophils depending on their localization (peri-alveolar or peri-sulcus), and the presence or not of *P. gingivalis*, strongly suggests that several subtypes of neutrophils are recruited or differentially matured in periodontitis. Their pro- or anti-inflammatory activity depends on the expression of several factors that define their function (49). The topographical distribution of neutrophils subsets and their function in periodontitis is still to be determined. As the role of NLRP3 in neutrophils is ill-defined (50), the importance of its overexpression in neutrophils near the alveolar bone crest will need further investigations to be deciphered.

## Conclusion

Taken together, our findings show that NLRP3 expression is protective against alveolar bone loss during traumatic inflammation of the periodontium. This protection possibly involves the targeting of neutrophils in the connective tissue, the functions of which may be disturbed by *P. gingivalis* whose presence deregulates NLRP3 expression in neutrophils close to the alveolar bone crest. The near absence of neutrophils in the gingival connective tissue of NLRP3 KO mice could be responsible for the withdrawal of protection leading to alveolar bone resorption. Our study strongly suggests that NLRP3 could be a switch to maintain/drive neutrophils within inflamed tissues. This effect is beneficial in the absence of *P. gingivalis*, promoting some protective activities of neutrophils. In contrast, the effect of NLRP3 could be detrimental in the presence of *the bacteria* which might activate the deleterious effector mechanisms of neutrophils.

## Data Availability Statement

The raw data supporting the conclusions of this article will be made available by the authors, without undue reservation.

## Ethics Statement

The animal study was reviewed and approved by the French Ministry of Higher Education, Research, and Innovation (approval APAFIS no. 2020032719365322).

## Author Contributions

BC: experiments, formal analysis. CT: experiments. AF: experiments. BB: resources. JS: experiments, software. LS: experiments, software. VW-S: writing. OH: conceptualization, writing. MG: supervision, conceptualization, validation, formal analysis, funding acquisition, writing—original draft. JB: supervision, conceptualization, validation, formal analysis, funding acquisition, writing—original draft. All authors contributed to the article and approved the submitted version.

## Funding

*In vivo* imaging was performed at the Life Imaging Facility of Paris Descartes University (Plateforme Imageries du Vivant by the FRM grant FRM DGE20111123012), supported by France Life Imaging (grant ANR-11- INBS-0006) and Infrastructures. URP2496 is affiliated to Laboratoire d’Excellence INFLAMEX (grant ANR-10-LABX-17). URP2496 is supported by the RHU iVASC (grant ANR-16-RHUS-0010_iVASC). This work was supported by contract grant sponsors: the French Society of Rheumatology (SFR), and the University of Paris (ANR-18-IDEX-0001, IdEx Université de Paris).

## Conflict of Interest

The authors declare that the research was conducted in the absence of any commercial or financial relationships that could be construed as a potential conflict of interest.

## Publisher’s Note

All claims expressed in this article are solely those of the authors and do not necessarily represent those of their affiliated organizations, or those of the publisher, the editors and the reviewers. Any product that may be evaluated in this article, or claim that may be made by its manufacturer, is not guaranteed or endorsed by the publisher.

## References

[1] Bostanci N Emingil G Saygan B Turkoglu O Atilla G Curtis MA. Expression and Regulation of the NALP3 Inflammasome Complex in Periodontal Diseases. Clin Exp Immunol (2009) 157:415–22. doi: 10.1111/j.1365-2249.2009.03972.x PMC2745037 19664151

[2] Shibata K. Historical Aspects of Studies on Roles of the Inflammasome in the Pathogenesis of Periodontal Diseases. Mol Oral Microbiol (2018) 33:203–11. doi: 10.1111/omi.12217 29360244

[3] Olsen I Yilmaz Ö. Modulation of Inflammasome Activity by Porphyromonas Gingivalis in Periodontitis and Associated Systemic Diseases. J Oral Microbiol (2016) 8:30385. doi: 10.3402/jom.v8.30385 26850450 PMC4744328

[4] Groslambert M Py B. Spotlight on the NLRP3 Inflammasome Pathway. J Inflamm Res (2018) 11:359–74. doi: 10.2147/jir.s141220 PMC6161739 30288079

[5] Kikuchi T Matsuguchi T Tsuboi N Mitani A Tanaka S Matsuoka M. Gene Expression of Osteoclast Differentiation Factor Is Induced by Lipopolysaccharide in Mouse Osteoblasts *via* Toll-Like Receptors. J Immunol (2001) 166:3574–9. doi: 10.4049/jimmunol.166.5.3574 11207318

[6] Duewell P Kono H Rayner KJ Sirois CM Vladimer G Bauernfeind FG. NLRP3 Inflammasomes are Required for Atherogenesis and Activated by Cholesterol Crystals. Nature (2010) 464:1357–61. doi: 10.1038/nature08938 PMC2946640 20428172

[7] Zambetti LP Mortellaro A. NLRPs, Microbiota, and Gut Homeostasis: Unravelling the Connection. J Pathol (2014) 233:321–30. doi: 10.1002/path.4357 24740681

[8] Xue F Shu R Xie Y. The Expression of NLRP3, NLRP1 and AIM2 in the Gingival Tissue of Periodontitis Patients: RT-PCR Study and Immunohistochemistry. Arch Oral Biol (2015) 60:948–58. doi: 10.1016/j.archoralbio.2015.03.005 25841070

[9] Huang X Yang X Ni J Xie B Liu Y Xuan D. Hyperglucose Contributes to Periodontitis: Involvement of the NLRP3 Pathway by Engaging the Innate Immunity of Oral Gingival Epithelium. J Periodontol (2015) 86:327–35. doi: 10.1902/jop.2014.140403 25325516

[10] Isaza-Guzmán DM Medina-Piedrahíta VM Gutiérrez-Henao C Tobón-Arroyave SI. Salivary Levels of NLRP3 Inflammasome-Related Proteins as Potential Biomarkers of Periodontal Clinical Status. J Periodontol (2017) 88:1329–38. doi: 10.1902/jop.2017.170244 28691886

[11] Kassebaum NJ Bernabé E Dahiya M Bhandari B Murray CJL Marcenes W. Global Burden of Severe Periodontitis in 1990-2010: A Systematic Review and Meta-Regression. J Dent Res (2014) 93:1045–53. doi: 10.1177/0022034514552491 PMC4293771 25261053

[12] Hajishengallis G Chavakis T. Local and Systemic Mechanisms Linking Periodontal Disease and Inflammatory Comorbidities. Nat Rev Immunol (2021) 21:426–40. doi: 10.1038/s41577-020-00488-6 PMC7841384 33510490

[13] Riep B Edesi-Neuß L Claessen F Skarabis H Ehmke B Flemmig TF. Are Putative Periodontal Pathogens Reliable Diagnostic Markers? J Clin Microbiol (2009) 47:1705–11. doi: 10.1128/JCM.01387-08 PMC2691128 19386852

[14] Lamont RJ Hajishengallis G. Polymicrobial Synergy and Dysbiosis in Inflammatory Disease. Trends Mol Med (2015) 21:172–83. doi: 10.1016/j.molmed.2014.11.004 PMC4352384 25498392

[15] Olsen I Lambris JD Hajishengallis G. Porphyromonas Gingivalis Disturbs Host-Commensal Homeostasis by Changing Complement Function. J Oral Microbiol (2017) 9:1340085. doi: 10.1080/20002297.2017.1340085 28748042 PMC5508361

[16] Gruber R. Osteoimmunology: Inflammatory Osteolysis and Regeneration of the Alveolar Bone. J Clin Periodontol (2019) 46(Suppl. 21):52–69. doi: 10.1111/jcpe.13056 30623453

[17] Graves DT Oates T Garlet GP. Review of Osteoimmunology and the Host Response in Endodontic and Periodontal Lesions. J Oral Microbiol (2011) 3:5304. doi: 10.3402/jom.v3i0.5304 PMC3087239 21547019

[18] Detzen L Cheat B Besbes A Hassan B Marchi V Baroukh B. NLRP3 Is Involved in Long Bone Edification and the Maturation of Osteogenic Cells. J Cell Physiol (2021) 236:4455–69. doi: 10.1002/jcp.30162 33319921

[19] Martinon F Pétrilli V Mayor A Tardivel A Tschopp J. Gout-Associated Uric Acid Crystals Activate the NALP3 Inflammasome. Nature (2006) 440:237–41. doi: 10.1038/nature04516 16407889

[20] Saadi-Thiers K Huck O Simonis P Tilly P Fabre J-E Tenenbaum H. Periodontal and Systemic Responses in Various Mice Models of Experimental Periodontitis: Respective Roles of Inflammation Duration and Porphyromonas Gingivalis Infection. J Periodontol (2013) 84:396–406. doi: 10.1902/jop.2012.110540 22655910

[21] Agnani G Tricot-Doleux S Du L Bonnaure-Mallet M. Adherence of Porphyromonas Gingivalis to Gingival Epithelial Cells: Modulation of Bacterial Protein Expression. Oral Microbiol Immunol (2000) 15:48–52. doi: 10.1034/j.1399-302x.2000.150108.x 11155164

[22] Bouxsein ML Boyd SK Christiansen BA Guldberg RE Jepsen KJ Müller R. Guidelines for Assessment of Bone Microstructure in Rodents Using Micro–Computed Tomography. J Bone Miner Res (2010) 25:1468–86. doi: 10.1002/jbmr.141 20533309

[23] Schindelin J Arganda-Carreras I Frise E Kaynig V Longair M Pietzsch T. Fiji: An Open-Source Platform for Biological-Image Analysis. Nat Methods (2012) 9:676–82. doi: 10.1038/nmeth.2019 PMC3855844 22743772

[24] Tsai W-H. Moment-Preserving Thresolding: A New Approach. Comput Vision Graph Image Process (1985) 29:377–93. doi: 10.1016/0734-189X(85)90133-1

[25] Costes SV Daelemans D Cho EH Dobbin Z Pavlakis G Lockett S. Automatic and Quantitative Measurement of Protein-Protein Colocalization in Live Cells. Biophys J (2004) 86:3993–4003. doi: 10.1529/biophysj.103.038422 15189895 PMC1304300

[26] Madel M-B Ibáñez L Wakkach A de Vries TJ Teti A Apparailly F. Immune Function and Diversity of Osteoclasts in Normal and Pathological Conditions. Front Immunol (2019) 10:1408. doi: 10.3389/fimmu.2019.01408 31275328 PMC6594198

[27] Inaoka T Bilbe G Ishibashi O Tezuka K Kumegawa M Kokubo T. Molecular Cloning of Human cDNA for Cathepsin K: Novel Cysteine Proteinase Predominantly Expressed in Bone. Biochem Biophys Res Commun (1995) 206:89–96. doi: 10.1006/bbrc.1995.1013 7818555

[28] Scott DA Krauss J. Neutrophils in Periodontal Inflammation. Front Oral Biol (2012) 15:56–83. doi: 10.1159/000329672 22142957 PMC3335266

[29] Kimura S Nagai A Onitsuka T Koga T Fujiwara T Kaya H. Induction of Experimental Periodontitis in Mice With Porphyromonas Gingivalis- Adhered Ligatures. J Periodontol (2000) 71:1167–73. doi: 10.1902/jop.2000.71.7.1167 10960025

[30] de Molon RS Mascarenhas VI de Avila ED Finoti LS Toffoli GB Spolidorio DMP. Long-Term Evaluation of Oral Gavage With Periodontopathogens or Ligature Induction of Experimental Periodontal Disease in Mice. Clin Oral Investig (2016) 20:1203–16. doi: 10.1007/s00784-015-1607-0 26411857

[31] Lin J Bi L Yu X Kawai T Taubman MA Shen B. Porphyromonas Gingivalis Exacerbates Ligature-Induced, RANKL-Dependent Alveolar Bone Resorption *via* Differential Regulation of Toll-Like Receptor 2 (TLR2) and TLR4. Infect Immun (2014) 82:4127–34. doi: 10.1128/IAI.02084-14 PMC4187858 25047844

[32] Rocha FRG Delitto AE de Souza JAC González-Maldonado LA Wallet SM Rossa Junior C. Relevance of Caspase-1 and Nlrp3 Inflammasome on Inflammatory Bone Resorption in A Murine Model of Periodontitis. Sci Rep (2020) 10:7823. doi: 10.1038/s41598-020-64685-y 32385413 PMC7210885

[33] Yamaguchi Y Kurita-Ochiai T Kobayashi R Suzuki T Ando T. Regulation of the NLRP3 Inflammasome in Porphyromonas Gingivalis-Accelerated Periodontal Disease. Inflamm Res (2017) 66:59–65. doi: 10.1007/s00011-016-0992-4 27665233

[34] de Molon RS de Avila ED Boas Nogueira AV Chaves de Souza JA Avila-Campos MJ de Andrade CR. Evaluation of the Host Response in Various Models of Induced Periodontal Disease in Mice. J Periodontol (2014) 85:465–77. doi: 10.1902/jop.2013.130225 23805811

[35] Chen Y Yang Q Lv C Chen Y Zhao W Li W. NLRP3 Regulates Alveolar Bone Loss in Ligature-Induced Periodontitis by Promoting Osteoclastic Differentiation. Cell Prolif (2021) 54:e12973. doi: 10.1111/cpr.12973 33382502 PMC7849172

[36] Lv K Wang G Shen C Zhang X Yao H. Role and Mechanism of the Nod-Like Receptor Family Pyrin Domain-Containing 3 Inflammasome in Oral Disease. Arch Oral Biol (2019) 97:1–11. doi: 10.1016/j.archoralbio.2018.10.003 30315987

[37] Inoue Y Shirasuna K Kimura H Usui F Kawashima A Karasawa T. NLRP3 Regulates Neutrophil Functions and Contributes to Hepatic Ischemia-Reperfusion Injury Independently of Inflammasomes. J Immunol (2014) 192:4342–51. doi: 10.4049/jimmunol.1302039 24696236

[38] Cheng R Wu Z Li M Shao M Hu T. Interleukin-1β Is a Potential Therapeutic Target for Periodontitis: A Narrative Review. Int J Oral Sci (2020) 12:1–9. doi: 10.1038/s41368-019-0068-8 31900383 PMC6949296

[39] Calkins CM Bensard DD Shames BD Pulido EJ Abraham E Fernandez N. IL-1 Regulates *In Vivo* C-X-C Chemokine Induction and Neutrophil Sequestration Following Endotoxemia. J Endotoxin Res (2002) 8:59–67. doi: 10.1177/09680519020080010601 11981446

[40] Cianciola LJ Genco RJ Patters MR Mckenna J Oss CJV. Defective Polymorphonuclear Leukocyte Function in a Human Periodontal Disease. Nature (1977) 265:445–7. doi: 10.1038/265445a0 834295

[41] Lavine WS Maderazo EG Stolman J Ward PA Cogen RB Greenblatt I. Impaired Neutrophil Chemotaxis in Patients With Juvenile and Rapidly Progressing Periodontitis. J Periodontal Res (1979) 14:10–9. doi: 10.1111/j.1600-0765.1979.tb00213.x 153958

[42] Ryder MI. Comparison of Neutrophil Functions in Aggressive and Chronic Periodontitis. Periodontol 2000 (2010) 53:124–37. doi: 10.1111/j.1600-0757.2009.00327.x 20403109

[43] Landzberg M Doering H Aboodi GM Tenenbaum HC Glogauer M. Quantifying Oral Inflammatory Load: Oral Neutrophil Counts in Periodontal Health and Disease. J Periodontal Res (2015) 50:330–6. doi: 10.1111/jre.12211 25040400

[44] Aboodi GM Goldberg MB Glogauer M. Refractory Periodontitis Population Characterized by a Hyperactive Oral Neutrophil Phenotype. J Periodontol (2011) 82:726–33. doi: 10.1902/jop.2010.100508 21080789

[45] Hajishengallis G Moutsopoulos NM Hajishengallis E Chavakis T. Immune and Regulatory Functions of Neutrophils in Inflammatory Bone Loss. Semin Immunol (2016) 28:146–58. doi: 10.1016/j.smim.2016.02.002 PMC4867283 26936034

[46] Silva LM Brenchley L Moutsopoulos NM. Primary Immunodeficiencies Reveal the Essential Role of Tissue Neutrophils in Periodontitis. Immunol Rev (2019) 287:226–35. doi: 10.1111/imr.12724 PMC7015146 30565245

[47] Allaeys I Rusu D Picard S Pouliot M Borgeat P Poubelle PE. Osteoblast Retraction Induced by Adherent Neutrophils Promotes Osteoclast Bone Resorption: Implication for Altered Bone Remodeling in Chronic Gout. Lab Invest (2011) 91:905–20. doi: 10.1038/labinvest.2011.46 21403645

[48] Chakravarti A Raquil M-A Tessier P Poubelle PE. Surface RANKL of Toll-Like Receptor 4–Stimulated Human Neutrophils Activates Osteoclastic Bone Resorption. Blood (2009) 114:1633–44. doi: 10.1182/blood-2008-09-178301 19546479

[49] Deniset JF Kubes P. Neutrophil Heterogeneity: Bona Fide Subsets or Polarization States? J Leuk Biol (2018) 103:829–38. doi: 10.1002/JLB.3RI0917-361R 29462505

[50] Tourneur L Witko-Sarsat V. Inflammasome Activation: Neutrophils Go Their Own Way. J Leukoc Biol (2019) 105:433–6. doi: 10.1002/JLB.3CE1118-433R 30720889

