# Supplementary material for: NLRP3 Is Involved in Neutrophil Mobilization in Experimental Periodontitis
Source: Front Immunol. 2022 Feb 23;13:839929. doi: 10.3389/fimmu.2022.839929 (PMC8905524; doi:10.3389/fimmu.2022.839929)
Supplement: Supplementary file 1 [file Image_1.pdf]

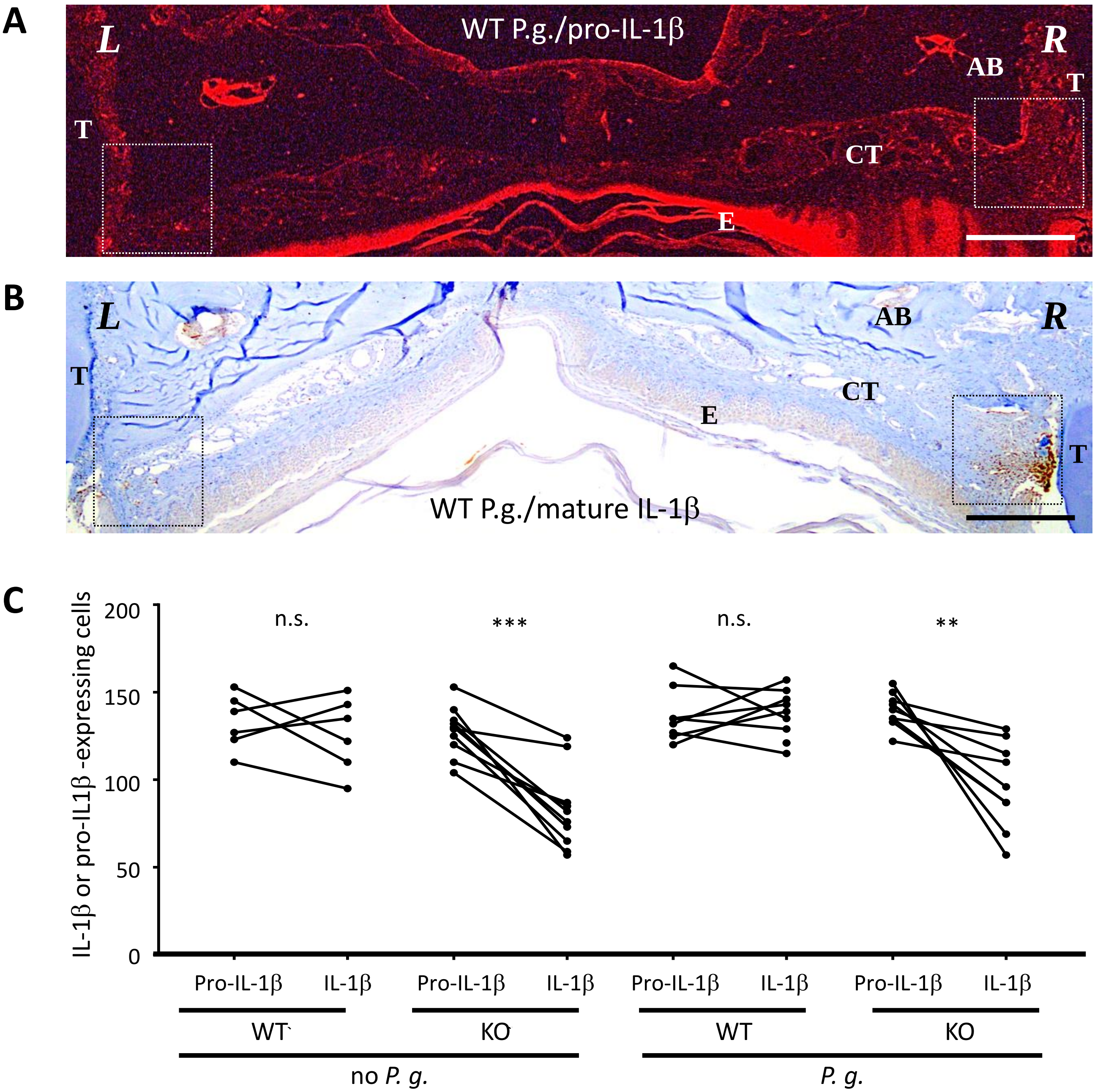

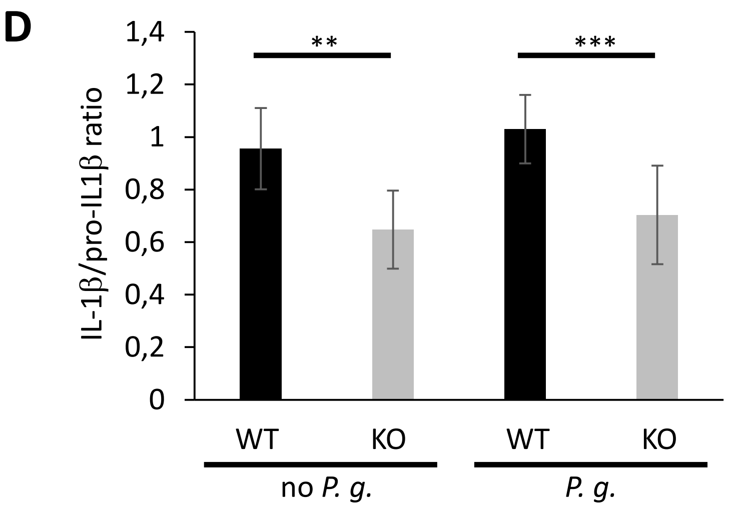

Supplementary Figure S1

Supp. Figure S1: Periodontal lesion and NLRP3 expression are required for optimal IL-1β maturation. (A, B) Whole palatine frontal sections of WT mice are shown, as the result of the assembly of two contiguous images. (L = left, R = right, T = tooth, E = epithelium, CT = connective tissue, AB = alveolar bone), bars = 500μm. **(C)** A correlative analysis between the number of cells expressing mature IL-1β and pro-IL-1β per mouse is shown. Ratio-paired T-test. **(D)** The ratio between the number of cells expressing mature IL-1β and pro-IL-1β was calculated. Histograms represent the mean +/- SD. Ordinary one-way ANOVA followed by uncorrected Fisher's LSD with a single pooled variance multiple comparison test. $^{**}P < 0.01$, $^{***}P < 0.001$, $^{n.s.}P \geq 0.05$, non-significant.
